# Data integrity issues: catalyst for a more robust approach to research on perioperative oxygen therapy?

**DOI:** 10.1186/s13741-019-0118-y

**Published:** 2019-07-04

**Authors:** Alex H. Oldman, Andrew F. Cumpstey, Daniel S. Martin, Michael P. W. Grocott

**Affiliations:** 1 Critical Care Research Group, Southampton National Institute of Health Research, Biomedical Research Centre, University Hospital Southampton, Southampton, UK; 2 Integrative Physiology and Critical Illness Group, Clinical and Experimental Sciences, Faculty of Medicine, University of Southampton, Southampton, UK; 3 Division of Surgery and Interventional Science (University College London) and Royal Free Perioperative Research Group, Department of Anaesthesia, Royal Free Hospital, 3rd Floor, Pond Street, London, NW3 2QG UK

**Keywords:** Oxygen, Perioperative care, Surgery, Anaesthetics, Hyperoxia

In February 2019, data integrity from 38 published randomised controlled trials in surgical patients conducted by an Italian surgical research group was called into question (Buranyi and Devlin 2019). Using the Carlisle method, Myles, Carlisle and Scarr identified evidence of compromised data in 38 out of 40 published articles from Mario Schietroma’s research group spanning from years 1993 to 2016 (Myles et al. 2019). In their analysis, they meta-analysed all published patient characteristic data, identifying poorly distributed participant characteristics, incorrect ‘*p*’ values and overly consistent results. There was enough evidence from this analysis to recommend an inquiry into the group’s work, and their published outputs are likely to have been misleading researchers within the field of perioperative medicine for some years (Myles et al. 2019). Such alleged misconduct is not only damaging to the scientific community and to perioperative research, but may also have substantial global consequences for patient safety through the impact of flawed research on doctors’ prescribing habits. Two of Schietroma’s papers had been referenced by the World Health Organisation’s (WHO) ‘Global guidelines for the prevention of surgical site infection’ and included in their meta-analysis on perioperative oxygenation published initially in 2016. In this, they made a strong recommendation (subsequently downgraded to ‘conditional’ in 2018) to provide 80% oxygen to all intubated patients undergoing surgery requiring general anaesthesia and intubation (Global guidelines for the prevention of surgical site infection 2018). To put this value into perspective, this is a recommendation to give surgical patients almost four times more inspired oxygen than is physiologically normal under healthy conditions.

The field of perioperative oxygen research is perplexing even for the well initiated. There seems to be a real contradiction between prescribing recommendations and trial evidence. Early studies reporting the beneficial effects of high-dose perioperative oxygen therapy in reducing surgical site infections and postoperative complications provided the majority of evidence for the 2016 WHO guidelines (Greif et al. 2000; Belda et al. 2005; Myles et al. 2007). These results, however, were not supported by data from a much larger trial, which showed no impact on surgical site infections from higher inspired oxygen concentrations (Meyhoff et al. 2009). Additionally, a Cochrane systematic review and meta-analysis published in 2015 concluded that there was insufficient evidence to support routinely giving anaesthetised patients more than 60% oxygen intraoperatively (Wetterslev et al. 2015). The most up-to-date systematic reviews and meta-analyses support the modified WHO recommendation, suggesting that there is “little evidence on safety-related issues” and “no definite signal of harm” with administering an 80% oxygen in adult patients undergoing general anaesthesia (de Jonge et al. 2019; Mattishent et al. 2019). These reviews support the fact that there remains little evidence for definite benefit of high perioperative fractions of oxygen and suggest that evidence for giving 80% oxygen in order to reduce the risk of surgical site infections has substantively changed, calling for the international recommendations to be urgently reconsidered (de Jonge et al. 2019).

Importantly, clinical outcome studies in patients in other contexts have provided evidence of harm from high-dose oxygen. Notably, the Improving Oxygen Therapy in Acute-illness (IOTA) systematic review, published in the Lancet in 2018, reported that liberal use of oxygen to maintain saturations above 96% was associated with increased in-hospital and 30-day mortality in acutely unwell patients (Chu et al. 2018). Whilst this study explicitly excluded patients undergoing elective surgery, observation of a harm signal across such a large and broad group of acutely unwell patients should be cause for concern for those advocating high-dose oxygen therapy in patients undergoing surgery. An additional concern that has more recently emerged from the literature is the potential adverse effect of high-dose perioperative oxygen therapy on longer-term outcomes. Such outcomes have also been studied across three follow-up analyses from the Danish PROXI trial (Fonnes et al. 2016; Meyhoff et al. 2012; Meyhoff et al. 2014), a randomised controlled trial of 1386 patients undergoing acute or elective laparotomy and given either 30% or 80% oxygen during and after surgery (Meyhoff et al. 2009). A 2-year post-hoc analysis of the PROXI data revealed that the incidence of acute MI was over twice as frequent in those patients given 80% oxygen in comparison to 30% (HR 2.86 (95% CI 1.10–7.44), *p* = 0.03) (Fonnes et al. 2016). When controlling for broader incidence of cardiac complications, researchers found statistical significance with higher fractions of oxygen and ‘any heart disease or death’ (HR 1.24 95% CI 1.06–1.45, *p* = < 0.01). An additional 2-year follow-up study demonstrated an increase in long-term mortality in cancer patients who received 80% oxygen (HR 1.45; 95% CI 1.10 to 1.90; *p* = 0.009) (Meyhoff et al. 2012), and, remarkably, a 4-year post-hoc study showed an overall reduction in cancer-free survival time across all trial patients receiving 80% oxygen (HR 1.19; 95% CI 1.01 to 1.42; *p* = 0.04) (Meyhoff et al. 2014). It is worth noting that these post-hoc analyses were not included in the WHO 2016 guidelines for SSI prevention. The PROXI trial was originally powered to detect a reduction in surgical site infections only, and therefore, these post-hoc analyses should still be treated with some caution; once an experimental group has been associated with one adverse outcome, it is not surprising that there might also be other adverse associations too. Despite this, their findings present an important new avenue for oxygen research and a starting point for hypotheses to be generated.

Paul Bert and James Lorrain Smith first described the detrimental effects of hyperbaric oxygen on the central nervous system and pulmonary tissues in the late nineteenth century (Martin and Grocott 2013). The spotlight has now moved to highlight the toxic effects of *normobaric* hyperoxia, which has profound effects on the cardiovascular system; causing increased systemic vascular resistance, reduced cardiac output, and coronary vasoconstriction, all of which limits coronary perfusion (Martin and Grocott 2013; Lumb and Walton 2012). Pulmonary tissue is also particularly susceptible to oxygen-induced injury, with dose-dependent pulmonary oedema, alveolar inflammation and atelectasis worsening ventilation/perfusion mismatching (Martin and Grocott 2013; Lumb and Walton 2012). Other organs, including the brain and kidneys are also susceptible to ‘hyperoxic injury’, and worse outcomes with hyperoxia are being reported in an increasing number of clinical scenarios; high-flow oxygen is no longer indicated in the management of acute coronary syndrome (particularly myocardial infarction) or neonatal resuscitation, despite previously being considered essential initial management in both situations (Cabello et al. 2016; Tan et al. 2005). Equally, clinical trials have shown increased harm with liberal oxygen therapy in stroke victims, critical illness and following cardiac arrest (Rincon et al. 2014; Diamani et al. 2014; Killgannon et al. 2010).

On a cellular level, these pathologies are likely mediated by reactive oxygen species (ROS)—unstable free radicals in part generated as by-products of oxidative phosphorylation in the mitochondrial electron transport chain (Helmerhost et al. 2015). ROS have many essential biological functions including oxygen sensing, cellular signalling and immune response modulation. However, excessive ROS build-up leads to oxidative stress, resulting in cellular damage through direct reactions with lipid membranes, DNA and proteins, inducing apoptosis and necrosis (Helmerhost et al. 2015; Auten and Davis 2009; Dias-Freitas et al. 2016). Hyperoxia appears to fuel this pro-inflammatory state, triggering cytokine cascades, neutrophil activation and worsening tissue oedema (Martin and Grocott 2013; Helmerhost et al. 2015). There is now an urgent need to delineate and quantify cellular responses to changes in oxygen availability in surgical patients and to determine how inspired oxygen fractions affect tissue inflammation and oxidative stress perioperatively.

The dissonance between oxygen guidelines and evidence from both clinical outcomes and mechanistic research is reflected in prescribing practices amongst UK anaesthetists. A recent multi-site evaluation of practice demonstrated that intraoperative oxygen administration by anaesthetists varied widely from 25 to 100%, with no obvious down-titration of oxygen in response to sustained supra-normal blood oxygen levels (Morkane et al. 2018). These findings were also supported by an observational cohort study of emergency department (ED) practice suggesting that clinicians may target hyperoxia in this context. The authors reported that acute hyper-oxygenation via an endotracheal tube in the ED was an independent predictor of hospital mortality (adjusted OR 1.95; 95% CI 1.34 to 2.85; *p* = < 0.001) (Page et al. 2018).

Despite a substantial number of high-quality cohort studies and clinical trials, there remains a paucity of high-quality evidence to demonstrate the physiological effects oxygen has during the perioperative period, particularly at a cellular level. Consequently, anaesthetic guidelines and recommendations for perioperative oxygen therapy currently remain poorly grounded in scientific understanding. We would argue that the revelations of the Myles, Carlisle and Scarr’s manuscript on the Schietroma papers should be used to provide perioperative physicians and anaesthetists with a fresh opportunity to discuss oxygen prescribing practices and promote high-quality research in order to clearly define safe and effective care in this area. Oxygen therapy remains *the* most common component of general anaesthesia, yet as a community, we appear to have little idea how much oxygen we should be prescribing for our patients and what pathophysiological mechanisms we should be basing this prescription on. This somewhat embarrassing paradox at the heart of anaesthetic practice requires urgent resolution if we are to reduce harm and improve the lives of those we care for during and after surgery.

## Data Availability

N/A
